# Antileishmanial Activity of *Schinus terebinthifolia* Essential Oil: Chemistry, In Vitro and Mechanistic Studies

**DOI:** 10.3390/molecules31071125

**Published:** 2026-03-29

**Authors:** Lianet Monzote, Lillyam Betancourt, Ramón Scull, Prabodh Satyal, Lizette Gil, Lars Gille, William N. Setzer

**Affiliations:** 1 Parasitology Department, Institute of Tropical Medicine “Pedro Kouri”, Havana 10400, Cuba; 2 Research Network Natural Products Against Neglected Diseases (ResNetNPND), 48149 Munster, Germany; 3 Pharmacological Research Department, Institute of Tropical Medicine “Pedro Kouri”, Havana 10400, Cuba; lillyam.betancourt@ipk.sld.cu (L.B.); lgil@ipk.sld.cu (L.G.); 4 Department of Pharmacy, Institute of Pharmacy and Food, Havana University, La Coronela, La Lisa, Havana 13600, Cuba; rscull@ifal.uh.cu; 5 Aromatic Plant Research Center, 230 N 1200 E, Suite 100, Lehi, UT 84043, USA; psatyal@aromaticplant.org; 6 Institute of Pharmacology and Toxicology, Department of Biomedical Sciences, University of Veterinary Medicine, Veterinärplatz 1, 1210 Vienna, Austria; lars.gille@vetmeduni.ac.at; 7 Department of Chemistry, University of Alabama in Huntsville, Huntsville, AL 35899, USA

**Keywords:** *Schinus terebinthifolia*, essential oil, protozoa, *Leishmania*, mitochondria, redox state

## Abstract

Leishmaniasis is caused by parasitic protozoans of the *Leishmania* genus and has been classified as a Neglected Tropical Disease. Control of this parasite relies mainly on chemotherapy; however, conventional available drugs are unsatisfactory. Phytomedicine, particularly essential oils, is a promising alternative. In this study, the chemical composition and antileishmanial properties of essential oil from leaves of *Schinus terebinthifolia* Raddi (EO-St) were determined. Chemical components were identified by GC-MS. Antileishmanial activity on promastigotes of *L. amazonensis* was assayed, followed by the evaluation of the essential oil’s effects on the mitochondrial membrane potential and redox state of the parasite. Finally, the activity was confirmed on intracellular amastigotes and compared with cytotoxicity on peritoneal macrophages from BALB/c mice. In the essential oil, 78 compounds were identified. The major component was δ-3-carene with 14.8%. The IC_50_ values of 18.2 ± 1.4 µg/mL and 15.0 ± 1.6 µg/mL against promastigote and amastigote forms, respectively, were obtained. The cytotoxicity for the host cells was approximately four-fold higher than those for the parasite. The essential oil was able to cause a disruption in the mitochondrial membrane potential. The quantified redox parameters showed statistical differences (*p* < 0.05) between EO-St-treated cultures and control groups (untreated and treated with DMSO). In summary, EO-St was active in vitro against both forms of *L. amazonensis*, possible mediated by mitochondrial dysfunction and redox imbalance.

## 1. Introduction

Neglected Tropical Diseases (NTDs) encompass a diverse group of diseases that have received limited attention and are prevalent in tropical areas worldwide. Leishmaniasis, an NTD, is a group of diseases that occur in humans and in other mammals in tropical and subtropical regions [1,2]. It is caused by more than 20 species of parasitic protozoans of the genus *Leishmania* and is transmitted by bites of sand flies from the Phlebotominae family. The disease manifests in several clinical forms, ranging from cutaneous leishmaniasis (CL), characterized by self-healing skin lesions, to potentially fatal visceral leishmaniasis (VL), depending on the infecting *Leishmania* species [3].

No vaccine for humans is available, and the control relies mainly on conventional therapies [4]. Pentavalent antimonial drugs are the first line of treatment, with other available alternatives, including amphotericin B (and liposomal formulations), pentamidine, miltefosine, and paromomycin. In general, these chemotherapies have specific limitations, such as high levels of toxicity, non-specificity for *Leishmania* parasites, treatment length leading to noncompliance, drug resistance that further complicates treatment efficacy, drug costs, and accessibility. Other challenges are related to variability in host genetics and immune response [5].

Due to the lack of effective and safe medicines and vaccines, it is crucial to explore alternative resources. Phytomedicine, which comprises therapeutic herbal constituents with anti-leishmanial properties, holds promise [2]. Within the group of plant-derived products, essential oils (EOs) are recognized to have a broad spectrum of interesting biological activities, including antileishmanial properties [6,7]. EOs are odorant products that could be extracted from different parts of plants and are generally composed of a mixture of different chemical compounds [8]. In particular, their physicochemical properties (volatility indicating small molecular size and lipophilicity) could allow for rapid diffusion through the cell membrane. This characteristic can enhance the targeting of EO active components to intracellular parasites [9], such as *Leishmania* parasites.

Recently, El-Nashar and collaborators highlighted that *Schinus* plants are rich sources of EOs. *Schinus terebinthifolia* Raddi (Anacardiaceae), popularly known as red pepper, is used as a gourmet spice in cooking, and some medicinal uses have been reported [10,11]. In these terms, antimicrobial, antioxidant, anti-inflammatory, antiparasitic, and antiviral properties have been summarized [10]. Although *S. terebinthifolia* has been one of the most extensively explored species in the *Schinus* genus, studies related to the chemical composition of plants growing in Cuba, as well as the antileishmanial activity of the EO from *S. terebinthifolia* (EO-St), have been scarcely studied.

**Figure 1:**
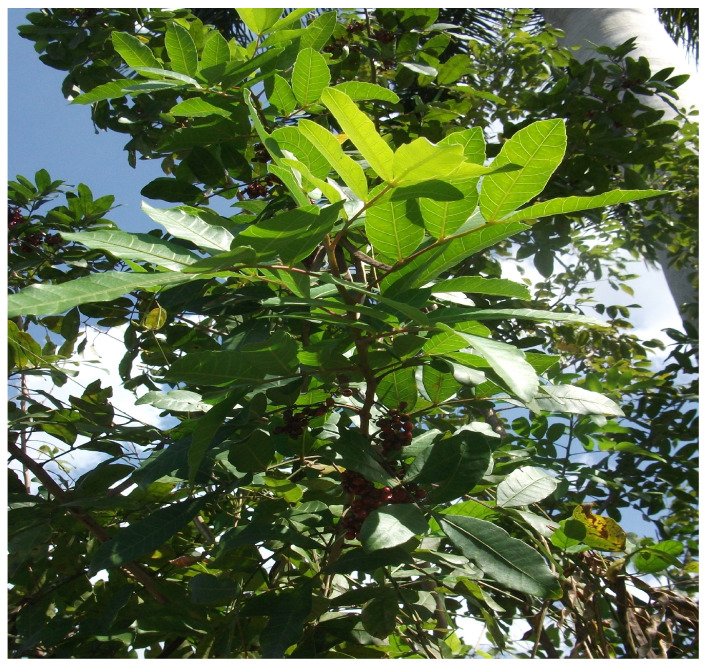
Photograph of *Schinus terebinthifolia* plant cultivated in the National Botanic Garden, Havana, Cuba (picture taken by the authors during the collection of the plant).

In the present work, the extraction and characterization of EO-St of Cuban plants (Figure 1) was determined. Additionally, the antileishmanial activity was assessed in vitro against *L. amazonensis* promastigote, and its influence on the mitochondrial membrane potential (MMP) and redox state of the parasite was evaluated. Finally, it was corroborated that EO-St is more active against intracellular parasites than against host macrophages.

## 2. Results and Discussion

The EO-St was obtained by hydrodistillation of the fresh leaves, yielding 0.7 ± 0.1%. The yield of leaf oil in this study was higher than those reported in Pakistan (0.09% [11]) or Egypt (0.5% [12]). In Brazil, however, studies show a high variation, from 0.63% in specimens collected in Umuarama [13] to 0.17% from São Paulo [14]. Also in Brazil, a recent study performed by Guimarães and collaborators [15] to evaluate the influence of seasonality on the yield of EO-St observed a ranged from 0.1% (July) to 0.7% (May and September), with an average of 0.5% during the year of investigation. In fact, reports found in the literature on the yield of EO-St reinforce the variability in the oil obtained from this species, which depend on plant characteristics, collection location, and genetic conditions [16]. Furthermore, in the present study, hydrodistillation was used to obtain the EO-St. This method is a simple technique that does not require complex or high-tech equipment, and only uses two elements (water and heat), which bring operational simplicity and low cost. Nevertheless, in the future, various extraction techniques could be compared in terms of efficiency, selectivity, yield, preservation of bioactive substances, chemical composition, and biological activity, including microwave-assisted hydrodistillation, microwave-assisted extraction, ultrasound-assisted extraction, supercritical CO_2_, and Soxhlet extraction [16,17]. In addition, these alternative extraction methods are also considered as promising extraction techniques due to their reduced extraction times, lower energy consumption, lower solvent requirement, and lower carbon dioxide emissions [17].

Gas chromatography with mass spectrometry (GC-MS) was used to study the EO-St obtained by hydrodistillation from the leaves by comparison of mass spectral data with the retention index (RI). The oil showed a very complex composition, with 78 compounds (Table 1), which constituted 85.9% of whole EO-St. Identified hydrocarbons and oxygenated terpenoids were 67.9 and 19.4%, respectively. The major chemical constituent was δ-3-carene with 14.8%, while α-pinene, *p*-cymene, β-phellandrene, *trans*-β-elemene, and γ-muurolene were around 6%. Unfortunately, 18 compounds were unidentified, with one of them (RI 1641) having a concentration of 6.1%. This limitation in chemical characterization could suggest the presence of new structures that have not been completely elucidated. Another previously studied Cuban essential oil presented similar characteristics [18].

The complexity and compound classes in the EO-St of this present study are consistent with previous reports, confirming large variations in qualitative and quantitative chemical compositions of EO-St collected from different geographical regions are noted [11,12,13,14,15,19,20,21,22,23,24,25,26,27,28,29]. Several examples of previous studies are summarized in Table 2. In general, the most abundant compound was α-pinene, which is present in the studied oil. However, other reported common compounds, such as limonene, (*E*)-β-caryophyllene, and germacrene D, are not present or only present in a small percent (<5%). It is interesting to note the unusual presence of δ-3-carene in a high percentage for the studied EO-St, which could suggest a different chemotype.

**Figure 2:**
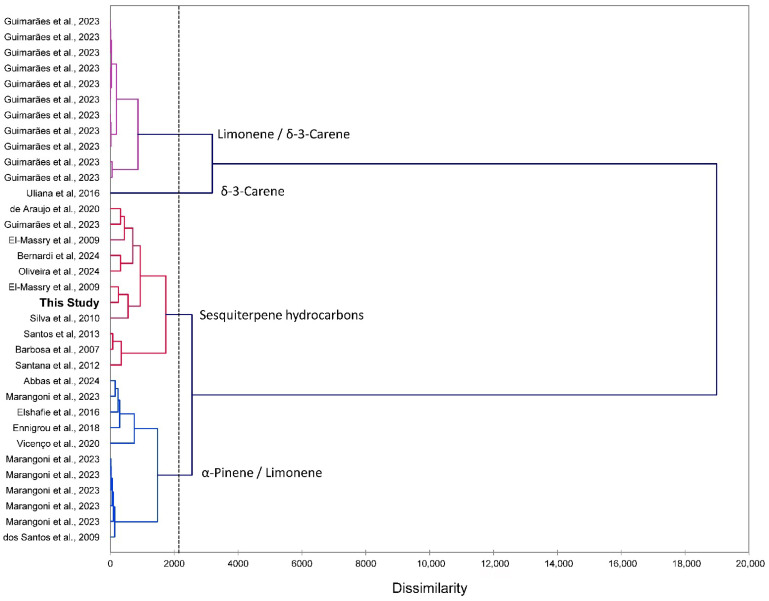
Dendrogram based on hierarchical cluster analysis (HCA) of the chemical compositions of *Schinus terebinthifolia* leaf essential oils collected in the National Botanic Garden, Havana, Cuba (**this study, bold**) and reported in the literature (by alphabetical order): Abbas et al., 2024 [11]; Barbosa et al., 2007 [22]; Bernardi et al., 2024 [27]; de Araújo et al., 2020 [24]; dos Santos et al., 2009 [26]; El-Massry et al., 2009 [12]; Elshafie et al., 2016 [21]; Ennigrou et al., 2018 [29]; Guimarães et al., 2023 [15]; Marangoni et al., 2023 [20]; Oliveira et al., 2024 [13]; Santana et al., 2012 [14]; Santos et al., 2013 [25]; Silva et al., 2010 [23]; Uliana et al., 2016 [28]; and Vicenço et al., 2020 [19].

**Figure 3:**
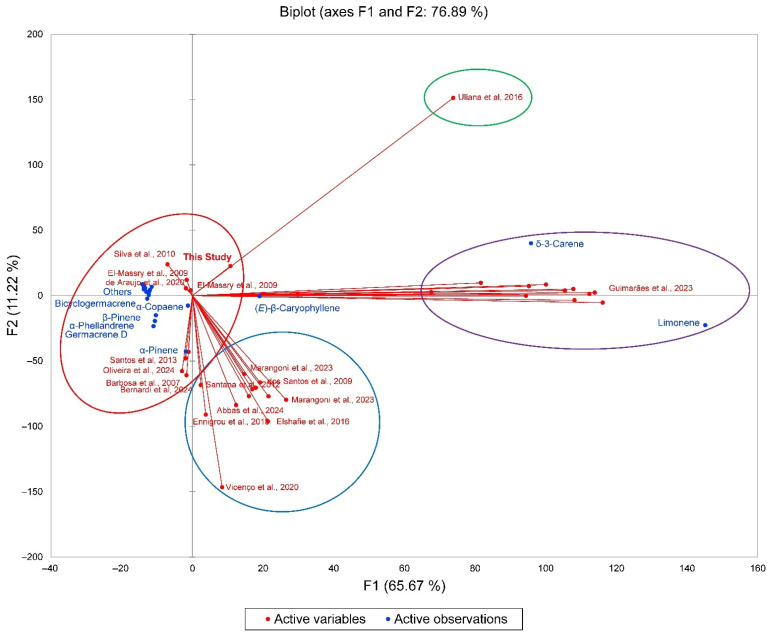
Biplot based on principal component analysis (PCA) of the chemical compositions of *Schinus terebinthifolia* leaf essential oils collected in the National Botanic Garden, Havana, Cuba (**this study, bold**) and reported in the literature (by alphabetical order): Abbas et al., 2024 [11]; Barbosa et al., 2007 [22]; Bernardi et al., 2024 [27]; de Araújo et al., 2020 [24]; dos Santos et al., 2009 [26]; El-Massry et al., 2009 [12]; Elshafie et al., 2016 [21]; Ennigrou et al., 2018 [29]; Guimarães et al., 2023 [15]; Marangoni et al., 2023 [20]; Oliveira et al., 2024 [13]; Santana et al., 2012 [14]; Santos et al., 2013 [25]; Silva et al., 2010 [23]; Uliana et al., 2016 [28]; and Vicenço et al., 2020 [19].

In order to place the *S. terebinthifolia* leaf essential oil composition from Cuba into context with EOs from other geographical locations [11,12,13,14,15,19,20,21,22,23,24,25,26,27,28,29], a hierarchical cluster analysis (HCA) was carried out using the percentages of the major essential oil components (Figure 2). The HCA can be described as four clusters, (i) a limonene/δ-3-carene chemotype, (ii) a δ-3-carene-rich chemotype with only one sample, (iii) a cluster made up of sesquiterpene-rich essential oils, and (iv) an α-pinene/limonene group, which illustrates the wide variation in essential oil composition for this species. The principal component analysis (PCA) corroborates the HCA, revealing the four clusters and their component correlations (Figure 3). The EO-St from Cuba shows a correlation with (*E*)-β-caryophyllene and δ-3-carene.

The plant is native to South America (western and southern Brazil, northeastern Argentina, and Paraguay) [30]. However, the plant has been introduced to the Caribbean region (including Cuba), southern North America, the Iberian peninsula, North Africa, tropical west Africa, southern Africa, southeast Asia, Australia, and Hawaii. Several essential oil samples from the native region are found in the α-pinene/limonene cluster. Interestingly, samples from Tunisia and Pakistan are also in the α-pinene/limonene cluster. However, the source of the original plant for these introduced plants was not indicated. The samples in the limonene/δ-3-carene cluster are from the Amazon region of Brazil. The sesquiterpene cluster is made up of samples from southern Brazil, western Brazil, Egypt, and Cuba (this sample). It is not clear that the composition differences are based on geographical location alone. Several factors may be responsible for variations in the chemical composition of essential oils within a species [31,32,33,34,35], including genetic factors, the abiotic environmental characteristics of the collection sites, seasonality/phenology, and biotic factors such as herbivory or fungal infection, as well as differences in processing methods.

**Figure 4:**
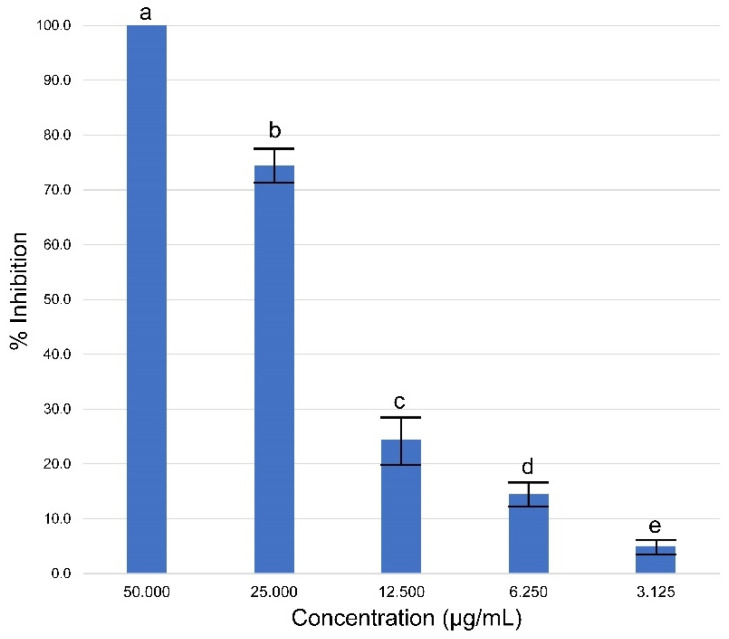
In vitro activity of essential oil from *Schinus terebinthifolia* against promastigotes of *Leishmania amazonensis*. Bars with the same letter are not significantly different at *p* ≤ 0.05.

The activity of EO-St against promastigotes of *L. amazonensis* was determined after 48 h of incubation at different concentrations (ranging 3.125 to 50 µg/mL), and viability was quantified using an MTT assay (Figure 4). A 100% growth inhibition of promastigotes at 50 µg/mL was observed, while 25 µg/mL caused a 74.4 ± 5.0% inhibition. Both concentrations (25 and 50 µg/mL) were statistically different (*p* < 0.05) compared to the rest of the tested concentrations (3.125, 6.25, and 12.5 µg/mL), which showed inhibition values lower than 25%. A median inhibitory concentration (IC_50_) of 18.2 ± 1.4 µg/mL was obtained.

To our knowledge, there are no reports on the antileishmanial activity of EO-St. However, previous studies on the antileishmanial activity of extracts from *S. terebinthifolia* against promastigotes of *L. amazonensis* gave contradictory results. While Moura-Costa and collaborators [36] did not observe the antileishmanial activity (IC_50_ > 200 µg/mL) of either aqueous and hydroalcoholic extracts from bark of *S. terebinthifolia* collected in Paraná (Brazil), Braga and collaborators [37] obtained an IC_50_ = 55 µg/mL of methanolic extract from the leaves and stem bark of plants from Minas Gerais (Brazil). In these previous studies, differences in the solvents used and collection locations could explain the observed biological differences. Furthermore, other factors could also be influential, including the time of harvest, the part of the plant used (whether stem or leaf), and the chemical characteristics of the extract/essential oil.

In addition, antimicrobial activity against a wide spectrum of bacteria (*Bacillus subtilis*, *Corynebacterium* sp., *Enterococcus feacium*, *Escherichia coli*, *Nocardia* sp., *Pseudomonas aeruginosa*, *Shigella dysenteriae*, *Staphylococcus albus*, *Staphylococcus aureus*, *Staphylococcus intermedius*, and *Streptococcus agalactiae*) and fungi (*Aspergillus niger*, *Aspergillus parasiticus*, and *Candida albicans*) have also been documented for EO-St [12,23,29,38].

The antileishmanial activity of essential oils and essential oil components has been reviewed [6]. Both δ-3-carene and α-pinene show antileishmanial activity against *L. amazonensis* promastigotes with IC_50_ values of 72.5 μg/mL and 19.7 μg/mL, respectively. Furthermore, α-pinene was active against the intracellular amastigote form of *L. amazonensis* (IC_50_ = 15.6 μg/mL). Additionally, (+)-α-pinene was shown to be the active enantiomer, with IC_50_ values of 36 μg/mL and 21 μg/mL on *L. amazonensis* promastigotes and amastigotes, respectively [39]. (*E*)-β-Caryophyllene also showed activity against *L. amazonensis* amastigotes (IC_50_ = 1.3 μg/mL) [6]. Consistent with these observations, commercial EOs rich in δ-3-carene and α-pinene have shown antileishmanial activity against *L. amazonensis* promastigotes [40]. For example, essential oil from *Abies sibirica* Leded (14.6% δ-3-carene, 15.2% α-pinene, IC_50_ 58.2 μg/mL), *Cupressus sempervirens* L. (27.0% δ-3-carene, 49.7% α-pinene, IC_50_ 40.0 μg/mL), and *Piper nigrum* L. (10.4% δ-3-carene, 11.1% α-pinene, IC_50_ 57.7 μg/mL) showed notable antileishmanial activity.

On the other hand, other species of *Schinus* displayed antiparasitic activity, including antileishmanial effects. A significant inhibition of *L. amazonensis* promastigote viability was caused by EO from leaves of *Schinus molle* L. collected in different Brazilian locations: IC_50_ = 21.4 μg/mL [41] and IC_50_ = 0.02 μg/mL [42].

Over the last few decades, studies regarding the antileishmanial activity of Cuban EOs have demonstrated in vitro activity on promastigotes of *L. amazonensis*. Some examples include EOs from *Artemisia absinthium* L. (IC_50_ = 14.4 µg/mL; [18]), *Croton linearis* Jacq. (IC_50_ = 20.0 µg/mL; [43]), *Dysphania ambrosioides* (L.) Mosyakin & Clemants (IC_50_ = 3.7 µg/mL; [44]), *Melaleuca leucadendra* (L.) L. (IC_50_ = 8.0 µg/mL; [45]), and *Pimenta dioica* L. (IC_50_ = 9.7 µg/mL; [46]). These findings demonstrate that the studied oil in this present work exhibited antileishmanial activity against promastigote forms in the same range (IC_50_ = 18.2 µg/mL), as previously reported in Cuban EOs (3.7 µg/mL < IC_50_ < 26.2 µg/mL).

**Figure 5:**
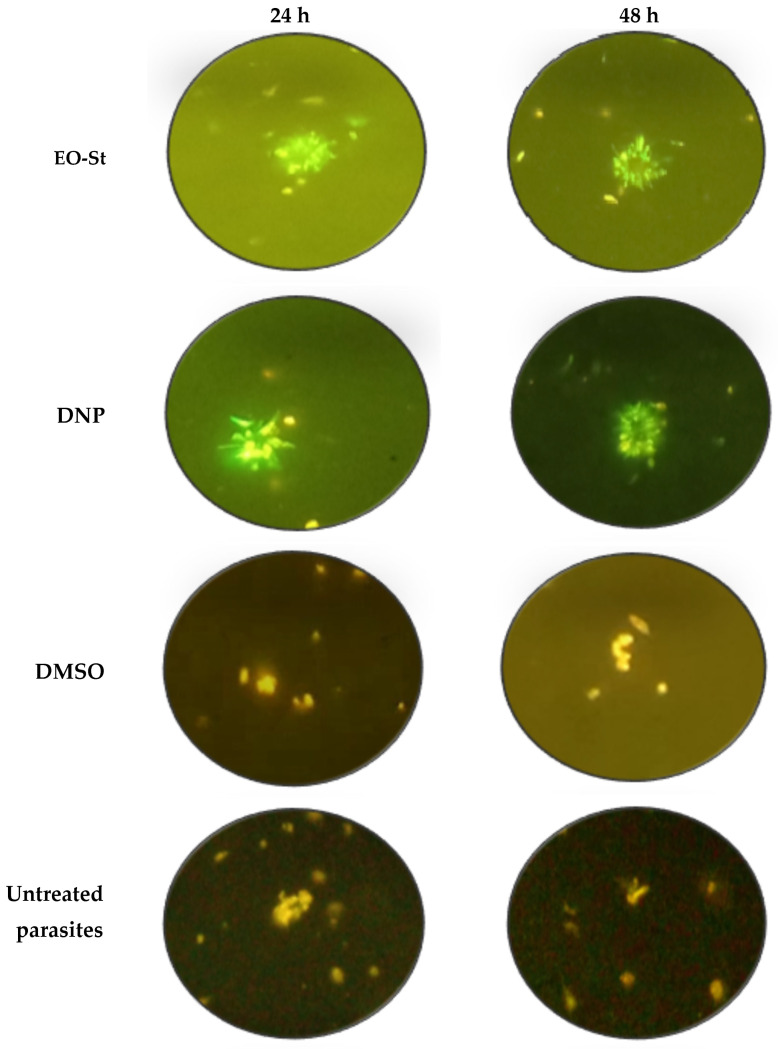
The influence of the essential oil from *Schinus terebinthifolia* on the mitochondrial membrane potential of *L. amazonensis* promastigotes assayed with JC-1 dye probe (400× magnification). Cultures of parasites (5 × 10^6^ promastigotes/mL) in RPMI medium were treated with 100 µg/mL of essential oil from *Schinus terebinthifolia* (EO-St), 2,4-dinitrophenol (DNP), dimethylsulfoxide (DMSO), or untreated parasites. The green color corresponds to JC-1 monomers (indicating a low mitochondrial membrane potential), and the orange color indicates JC-1 aggregates (indicating a high mitochondrial membrane potential).

The action of EO-St on mitochondrial membrane potential (MMP) was explored, since other EOs have also been shown to exert their antileishmanial action through interference with mitochondrial function, including the EO from *D. ambrosioides* [44] and *Tagetes lucida* Cav. [47]. Furthermore, *S. molle* essential oil caused MMP disruption in treated *L. amazonensis* [42]. In the present study, MMP was assayed using JC-1 dye, which is a cationic mitochondrial vital dye that becomes concentrated in the mitochondria in proportion to its variation in MMP- and ATP-generating capacity [48]. In this case, red fluorescence (JC-1 aggregates) demonstrated normal MMP, while green fluorescence corresponds to low MMP. The changes in fluorescence (from orange to green) suggest a breakdown of MMP in response of a mitochondrial dysfunction. In this study, EO-St cause a green predominant fluorescence in promastigotes of *L. amazonensis* treated during 24 h or 48 h at a concentration of 100 µg/mL (Figure 5), which suggest a disruption of MMP. Similarly, green fluorescence was observed in parasites treated with 2,4-dinitrophenol (DNP), a conventional uncoupler used as positive control, while the negative control (parasite treated with dimethylsulfoxide (DMSO), EO-St vehicle) and untreated promastigotes showed red fluorescence.

**Figure 6:**
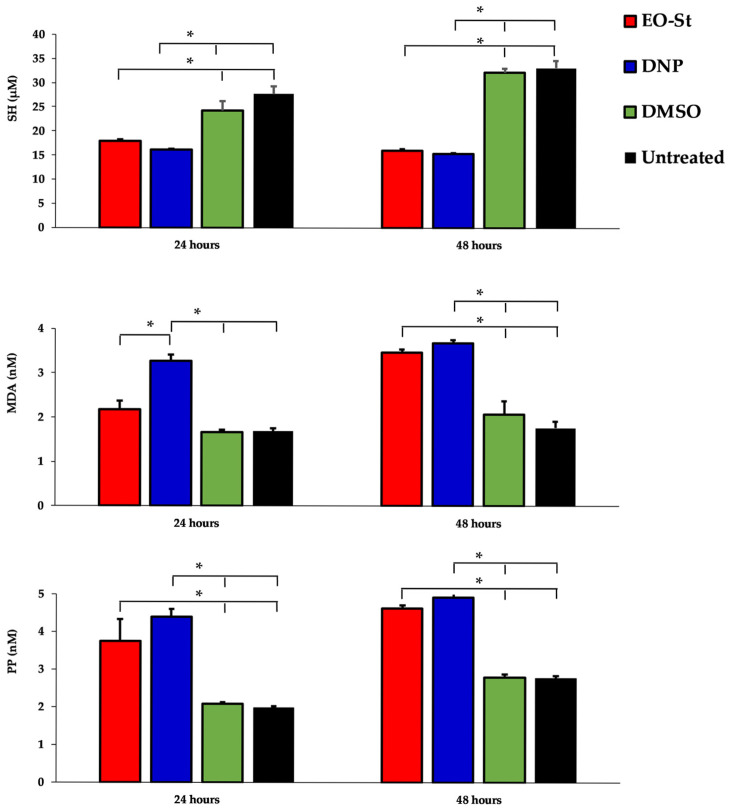
Redox indices in the supernatant of cultures of promastigotes of *Leishmania amazonensis* treated with 100 µg/mL of the essential oil from *Schinus terebinthifolia* (EO-St), 2,4-dinitrophenol (DNP), dimethylsulfoxide (DMSO) during 24 or 48 h, or untreated parasites. SH: Sulfhydryl groups (SH); MDA: malondialdehyde groups; PP: peroxidation potential: * Statistical differences with *p* < 0.05.

In addition, the redox state of treated *L. amazonensis* promastigotes with EO-St was also assayed under the same conditions (treatment of 24 h or 48 h at 100 µg/mL). In this sense, sulfhydryl groups (SH), malondialdehyde groups (MDA), and the potential of peroxidation (PP) were determined. In general (Figure 6), the quantified redox parameters showed statistical differences (*p* < 0.05) between treated (with EO-St and DNP) and control groups (treated with DMSO and untreated). Notably, EO-St treatment displayed statistically equivalent concentrations of SH and PP (*p* > 0.05) compared to cultures treated with positive control DNP after 24 or 48 h of incubation. However, in the MDA case, after 24 h, the cultures treated with EO-St did not reach the concentration caused by DNP (*p* < 0.05), but after 48 h of incubation, the concentrations of both groups (treated with EO-St and DNP) were comparable (*p* > 0.05).

The decrease in SH groups and concomitant increase in MDA and PP suggest a state of oxidative stress and the inability of antioxidant systems (in both lipid and aqueous phases) in parasites to compensate for these reactive oxygen species (ROS). These alterations of redox indices detected in the treated cell could be a consequence of the ROS generation related to mitochondrial dysfunction due to the loss of MMP, which causes the death of parasites. The observed cellular mechanism of action aligns with the activity of other terpenoid-rich essential oils reported in the literature, in which the antileishmanial activity could also be linked to the capacity of an oil to interact with mitochondrial membranes by generating free radicals that oxidize parasite macromolecules, leading ultimately to its death by apoptosis or necrosis. In fact, the antileishmanial activity of some studied EOs could probably be attributed to the lipophilic terpenes that could function as uncouplers [49,50,51].

Finally, to corroborate the antileishmanial potency of EO-St, an evaluation against intracellular amastigotes of *L. amazonensis* was carried out after 48 h of incubation. In parallel, a cytotoxicity assay was performed against non-infected peritoneal macrophages from BALB/c mice. In both cases, incubation with drugs was over a 48-h period, and Pentamidine^®^ was used as positive control.

The results presented in Table 3 show that EO-St maintained antileishmanial activity with the same IC_50_ (*p* > 0.05), compared to the promastigote form (IC_50_ = 18.2 µg/mL). Therefore, the antileishmanial activity of EO-St probably depends on proper parasite function in both forms. In the scientific literature, we did not find any previous reports about the activity of EO-St on intracellular amastigotes of *L. amazonensis*. Morais and collaborators [52] reported that a *n*-hexane extract from *S. terebinthifolia* leaves caused leishmanicidal activity against amastigotes of *L. infantum*, with IC_50_ values between 28 and 97 µg/mL. In the studied extract, a preponderant percent of terpenoid compounds were identified, which could explain, in part, the observed antileishmanial activity. Other publications support this hypothesis, since terpenoids have been identified as potent antiparasitic compounds, including antileishmanial activity [53]. For example, Santana and collaborators [54] reported an IC_50_ of 37 µg/mL of α-pinene against intracellular amastigotes of *L. amazonensis*, while Capello and collaborators [55] showed that β-elemene reduced parasitism and eliminated the majority of infected J774 macrophages with *L. amazonensis* cells at 50 µg/mL. Recently, an in silico molecular docking analysis published by Barbosa and collaborators [56] showed that γ-muurolene presented favorable affinity energy values (around −7 kcal/mol) for three *Leishmania* key enzymes: dihydroorotate dehydrogenase, sterol 14α-demethylase, and trypanothione reductase.

To evaluate the potential therapeutic benefit of EO-St, the antileishmanial activity and cytotoxicity to host cells were compared. This comparison helps us to determine the therapeutic margin or the effective dose against the pathogen without harmful effects on the host. Low selectivity could indicate systemic toxicity, making the compound unsuitable for therapeutic use. In this study, the EO-St IC_50_ was lower than five times, which is the international criterion defined for Caridha and collaborators [57] for products against CL. This is important for several scientific and applicative reasons. However, for cutaneous leishmaniasis to be feasible, a local formulation can alleviate the systemic toxicity of the product. With this finding, in our laboratory, EOs from different Cuban plants were been evaluated by the intralesional route, which have demonstrated antileishmanial efficacy and good tolerance by BALB/c mice. EOs from *A. absinthium* [18], *M. leucadendra* [45], and *P. dioica* [46] are examples in this respect. With this finding, topical application could be an attractive route of administration, not only to reduce side effects, but also to avoid the injectable route, the first-pass metabolism, and pre-systemic elimination within the gastrointestinal tract. In particular, evidence of penetration into and across the skin of EOs to exert local therapeutic actions have been shown [58].

## 3. Materials and Methods

### 3.1. Plant and Essential Oils

Aerial parts of *S. terebinthifolia* plants were collected during the early hours of the morning in March at the National Botany Garden (NBG), Havana, Cuba. A specimen was deposited at the Herbarium of Cuban Flora of the NBG and authenticated by Ph.D. Eldys Bécquer (voucher number: 8501918). Leaves of collected samples were selected, rinsed with abundant water, and manually crushed into small pieces. Then the fresh leaf material was conventionally hydrodistilled using a Clevenger-type apparatus for 3 h after the water commenced boiling, and the EO-St was obtained. The yield (% *w*/*w*) of EO-St was obtained by essential oil mass (g)/plant mass (g) × 100, considering fresh leaves [13].

The EO-St was chemically characterized by GC-MS using a Shimadzu GCMS-QP2010 Ultra GC-MS instrument (Shimadzu Scientific Instruments, Columbia, MD, USA). A 100-μL sample of the essential oil was diluted with CH_2_Cl_2_ to give a 5% *w*/*v* solution, and a 0.1 μL sample was injected. The splitting mode was 30:1; there was a ZB-5-fused silica capillary column with a length of 30 m and an internal diameter of 0.25 mm, a (5% phenyl)-polymethylsiloxane stationary phase, and a film thickness of 0.25 μm (Phenomenex, Torrance, CA, USA). Helium was the carrier gas, with a column head pressure of 552 kPa and a flow rate of 1.37 mL/min. The injector temperature was 250 °C, and the ion source temperature was 200 °C. The GC-MS instrument was operated in electron impact (EI) mode (electron energy = 70 eV); the scan range was 40–400 atomic mass units, and the scan rate was 3.0 scans/s. The GC oven temperature program was set to 50 °C as the initial temperature, which was increased at a rate of 2 °C/min until a final temperature of 260 °C. Finally, identification of the oil components was based on their RI, determined by reference to a homologous series of *n*-alkanes, and by comparison of their mass spectral fragmentation patterns with those reported in the available databases: Adams [59], FFNSC3 [60], NIST20 [61], and Satyal [62].

### 3.2. Parasites and Cells

The reference strain MHOM/77BR/LTB0016 of *L. amazonensis* was used for screening. Parasites were obtained by aspiration with a syringe from mouse lesions, and cultivated at 26 °C in Schneider’s medium (Sigma-Aldrich, St. Louis, MO, USA) supplemented with 10% heat-inactivated fetal bovine serum (HFBS; Sigma-Aldrich, St. Louis, MO, USA) and antibiotics solution (100 U of penicillin, 100 μg of streptomycin, Sigma-Aldrich). When the promastigotes were discernible, subsequent passages were carried out every 3 or 5 days to obtain subcultures for the experiments.

Normal female BALB/c mice were used to obtain murine peritoneal macrophages (MPM), the host cell of amastigotes of *Leishmania*. Mice were purchase from the National Center of Laboratory Animals Production (CENPALAB, Havana, Cuba), including their certificate of quality assurance. The mice were maintained under standard laboratory conditions, with food and water available ad libitum during the study. The experimental procedures were carried out according to the Guide for the Care and Use of Laboratory Animals (Eighth Edition). The experiments were approved by the Ethics Committee (CEI-IPK 44-20), Havana, Cuba. Peritoneal macrophages were obtained from mouse peritoneum, and were washed with RPMI medium (Sigma, St. Louis, MO, USA) and antibiotics (100 μg of streptomycin/mL and 100 U of penicillin/mL; Sigma, St. Louis, MO, USA) at the moment of use.

### 3.3. Activity of EO-St Against Promastigotes of L. amazonensis

The antipromastigote viability test was carried using a 96-well plate format, as previously reported [46]. Briefly, the medium (100 μL of Schneider’s medium + HFBS + antibiotics) was distributed in each well. In the last lane of the plate, 4 μL of EO-St stock solution and 96 μL of medium were added (the final concentration of DMSO was 1%). Then, five 1:2 serial dilutions were performed, and 100 µL of parasites suspended in the exponentially growing phase was then added (the final concentration of parasites was 10^6^ promastigotes/mL). The plates were then sealed with Parafilm^®^ and incubated at 26 °C for 72 h. Lane A was used as the medium control (medium without parasites) and lane B constituted the negative control (medium with parasites without test samples). After 72 h incubation, 10 μL of 3 mM resazurin solution (Sigma-Aldrich, St. Louis, MO, USA) was added to each well, and the plates were incubated for an additional 4 h. The absorbance was determined with a Molecular Devices plate reader (San Jose, CA, USA) with a test wavelength of 560 nm and a reference wavelength of 600 nm. The IC_50_ was calculated from linear dose–response curves fit to the data using a linear equation model. Each concentration of products was carried out in triplicate, and experiments were repeated three times. The results are expressed as means ± SD.

### 3.4. Variation in MMP in L. amazonensis Treated with EO-St

The variation in MMP was evaluated using JC-1 (5,5′,6,6′-Tetrachloro-1,1′,3,3′-tetraethylbenzimidazolylcarbocyanine chloride; Biotium, Hayward, CA, USA) dye as a probe [63]. Briefly, a culture of *L. amazonensis* promastigotes in the exponential growth phase was adjusted at 10^6^ parasites/mL and treated with 100 µg/mL of EO-St and 200 µM of DNP or DMSO (0.5%) over 24 h and 48 h periods at 26 °C. After the incubation periods, parasites were collected, concentrated, and then incubated for 10 min with 10 µM of JC-1. The fluorescence was observed using a Leitz Wetzlar (Wetzlar, Germany) fluorescence microscope at 400×. Photographs of the different analyses were obtained.

### 3.5. Determination of REDOX Status of Promastigotes of L. amazonensis Treated with EO-St

Promastigots of *L. amazonensis* in the exponential growth phase was adjusted to 10^6^ parasites/mL and treated with 100 µg/mL of EO-St, 200 µM of DNP, or DMSO (0.5%) over 24 h and 48 h periods at 26 °C. After the incubation periods, the cultures were centrifugated for 10 min at 170× *g*, and the supernatant was collected to determine SH, PP, and MDA.

Protein sulfhydryl groups were analyzed with the method described by Sedlak and Lindsay [64]. SHT (Glutathione; Sigma, St. Louis, MO, USA) was used to generate standard curves. The absorbances were obtained at a wavelength of 412 nm using a Zuzi Spectrophotometer (Auxilab, Navarra, Spain). MDA concentrations were determined using the LPO-586 kit (Calbiochem, La Jolla, CA, USA) and the chromogenic reagent *N*-methyl-2-phenylindole. Before the assay, BHT (0.01% (*v*/*v*) of a 2% stock solution in ethanol) and EDTA (final concentration of 1 µM) were added to the sample to avoid lipid oxidation. The absorbances of formed chromophore were obtained after 40 min of incubation at 45 °C at a wavelength of 586 nm using a Pharmacia Spectrophotometer. Freshly prepared solutions of MDA bis[dimethylacetal] (Sigma, St. Louis, MO, USA) were used as reference standards and were assayed under identical conditions [65,66]. The PP was calculated by subtracting the MDA concentrations at time 0 from the one obtained after 24 h or 48 h [67].

### 3.6. Activity of EO-St Against Amastigotes of L. amazonensis, and Cytotoxicity Against Peritoneal Macrophages from BALB/c Mice

For antileishmanial activity, MPM was obtained from healthy BALB/c mice by peritoneal washing with RPMI medium (Sigma, St. Louis, MO, USA) supplemented with antibiotics and plated at 10^6^ cells/mL in a 24-well plate. After an incubation at 37 °C and 5% CO_2_ during a 2 h period, non-adherent cells were removed and stationary-phase *L. amazonensis* promastigotes were added at a 4:1 parasite/macrophage ratio in medium supplemented with heat-inactivated fetal bovine serum (HFBS; Sigma-Aldrich, St. Louis, MO, USA). The plate was incubated at 37 °C and 5% CO_2_ for 4 h, after which the free parasites were also removed. Subsequently, test products were added, and four serial dilutions 1:2 were carried out. The plate was incubated under the same conditions for 48 h [68]. After that, the supernatant was removed, the cells were fixed with methanol, stained with 10% Giemsa, and examined microscopically (Motic, Hong Kong, China) under immersion oil at 1000×. The total parasite burden was determined according to the number of infected macrophages and the number of amastigotes occupying the macrophages by counting 100 macrophages [69]. IC_50_ values were calculated from linear dose–response curves fit to the data using a linear equation model. The experiments were repeated three times, and the results are expressed as means ± SD.

Macrophages from BALB/c were obtained by peritoneal washing in RPMI medium and antibiotics, as previously mentioned, and were seeded in 96-well plates at 3 × 10^5^ cells/well. The plates were incubated at 37 °C and 5% CO_2_ over 2 h, and the non-attached cells were removed. Then, fresh medium with HFBS and EO-St or pentamidine at different concentrations were added, and incubation continued for an additional 48 h. Cellular viability was then determined using MTT (3-[4,5-dimethylthiazol-2-yl]-2,5-diphenyltetrazolium bromide, Sigma, St. Louis, MO, USA). MTT solutions were prepared at 5 mg/mL in saline solution, filtered and sterilized immediately prior to use, and 15 µL was added to each well. After incubation for an additional 4 h, the formazan crystals were dissolved with 100 µL of DMSO. The optical density was determined using an EMS Reader MF Version 2.4-0 at a test wavelength of 560 nm and a reference wavelength of 630 nm [70]. CC_50_ were calculated from linear dose–response curves, and the results are expressed as the means ± SD of three replicates.

Finally, Selectivity Index (SI) values were calculated: SI = CC_50_ (macrophages)/IC_50_ (*L. amazonensis* amastigotes).

### 3.7. Statistical Analysis

The antileishmanial activity for EO-St on *L. amazonensis* promastigotes was analyzed using three replicate trials for each concentration. The data are expressed a means ± standard deviations. Analysis of variance was conducted by one-way ANOVA followed by the Tukey test using Minitab^®^ 22.4 (Minitab Inc., State College, PA, USA). Comparisons between the test samples and the negative control sample, in triplicate, for the redox indices were performed by ANOVA with Games–Howell post-test using software SPSS version 20. In all cases, statistical significant differences were identified for *p* < 0.05. The HCA and the PCA were carried out using XLSTAT v. 2018.1.1.62926 (Addinsoft, Paris, France) of the studied EO-St with those reported in the literature [11,12,13,14,15,19,20,21,22,23,24,25,26,27,28,29]. The concentrations (percentages) of the most abundant essential oil constituents (α-pinene, β-pinene, α-phellandrene, *iso*-sylvestrene, δ-3-carene, *p*-cymene, limonene, β-phellandrene, *cis*-β-terpineol, citronellal, verbenone, carvone, *p*-cymen-7-ol, α-copaene, *trans*-β-elemene, β-longipinene, (*E*)-β-caryophyllene, β-cedrene, aromadendrene, 9-*epi*-(*E*)-caryophyllene, γ-gurjunene, germacrene D, bicyclogermacrene, and spathulenol) were used for both analyses. Dissimilarity was used for cluster determination based on the Euclidean distance, and Ward’s method was used to define agglomeration. The PCA, type covariance, was used to verify the results of the HCA.

## 4. Conclusions

To the best of our knowledge, EO-St was analyzed in this study for the first time for its antileishmanial potential. Chemical characterization showed an unusual composition in which δ-3-carene was the main compound. Antileishmanial activity was demonstrated in vitro against *L. amazonensis* promastigote, probably due to mitochondrial dysfunction and the unbalance of the redox state of the parasite. Future investigations should focus on (i) designing local formulations with EO-St to validate these findings in vivo and to optimize its use in pharmaceutical settings, or (ii) exploring the effect of pure compounds and their synergistic effects to eliminate the cytotoxic components and develop an alternative based on terpenoid compounds. EO-St, or pure terpene compounds, could be another product based on an EOs that could be explored in the search for therapeutic alternatives for leishmaniasis, addressing the pressing need for an accesible and efficient product.

## Figures and Tables

**Table 1:** Peak assignment for gas chromatography–mass spectrometry profiles of the essential oil extracted by hydrodistillation from leaves of *Schinus terebinthifolia* collected in the National Botanic Garden, Havana, Cuba.

RI_calc_	RI_db_	Compounds	%	RI_calc_	RI_db_	Compounds	%
927	930	α-Thujene	0.2	1382	1388	β-Bourbonene	0.2
**942**	**939**	**α-Pinene**	**6.1**	1385	1383	*cis*-β-Elemene	0.2
953	954	Camphene	0.1	**1392**	**1390**	***trans*-β-Elemene**	**6.3**
972	971	3,7,7-Trimethylcyclohepta-1,3,5-triene	0.1	1408	1408	*cis*-5-Hydroxy-*p*-menth-6-en-2-one	0.1
975	975	Sabinene	0.2	1416	1419	(*E*)-β-Caryophyllene	2.2
977	979	β-Pinene	0.6	1425	1417	*trans*-5-Hydroxy-*p*-menth-6-en-2-one	0.6
992	990	Myrcene	1.2	1433	1436	γ-Elemene	0.1
1003	1002	α-Phellandrene	3.2	1436	1441	Aromadendrene	0.1
**1009**	**1011**	**δ-3-Carene**	**14.8**	1442	1444	6,9-Guaiadiene	0.1
1015	1017	α-Terpinene	0.1	1444	1442	(*Z*)-β-Farnesene	0.1
1022	1024	*m*-Cymene	0.1	1447	1448	*cis*-Muurola-3,5-diene	0.3
**1025**	**1026**	***p*-Cymene**	**6.3**	1450	1452	α-Humulene	0.3
**1027**	**1029**	**β-Phellandrene**	**6.7**	1460	1456	(*E*)-β-Farnesene	0.2
1028	1031	1,8-Cineole	1.0	1476	1476	*trans*-Cadina-1(6),4-diene	0.4
1048	1050	(*E*)-β-Ocimene	tr	**1480**	**1479**	**γ-Muurolene**	**6.8**
1057	1059	γ-Terpinene	0.1	1484	1480	*ar*-Curcumene	1.5
1085	1187	p-Mentha-1,7(8)-dien-2-ol	0.0	1494	1498	α-Selinene	0.5
1087	1088	Terpinolene	0.3	1495	1496	Bicyclogermacrene	0.2
1120	1118	*cis-p*-Menth-2-en-1-ol	0.1	1501	1500	α-Muurolene	0.3
1122	1115	Cyclooctanone	0.1	1503	1500	Isodaucene	1.8
1135	1137	*cis-p*-Mentha2,8-dien-1-ol	0.1	1511	1505	β-Bisabolene	0.3
1137	1139	*trans*-Pinocarveol	0.1	1514	1513	γ-Cadinene	0.4
1139	1139	*trans-p*-Menth-2-en-1-ol	0.1	1517	1515	(Z)-γ-Bisabolene	1.1
1143	1146	Camphor	tr	1524	1523	δ-Cadinene	1.1
1144	1144	*trans*-Verbenol	tr	1555	1561	Germacrene B	1.4
1165	1669	Borneol	tr	1577	1578	Spathulenol	2.6
1177	1177	Terpinen-4-ol	0.5	1582	1583	Caryophyllene oxide	0.5
1184	1182	*p*-Cymen-8-ol	0.1	1582	1590	Globulol	0.7
1184	1182	*p*-Methylacetophenone	0.1	1590	1592	Viridiflorol	0.1
1186	1185	Cryptone	0.6	1593	1594	Salvial-4(14)-en-1-one	0.4
1192	1188	α-Terpineol	0.4	1631	1629	*iso*-Spathulenol	2.0
1200	1196	Methyl chavicol (=Estragole)	0.6	1641	---	Unidentified ^b^	6.1
1202	1202	*cis*-Sabinol	0.5	1642	1641	τ-Cadinol	0.4
1207	1190	*cis*-Chrysanthenyl formate	0.9	1644	1643	τ-Muurolol	0.4
1209	1211	*trans*-Piperitol	0.2	1649	1646	α-Muurolol	0.2
1251	1248	Car-3-en-2-one	0.2	1655	1653	Pogostol	0.7
1254	---	5-Isopropyl-1-methyl-3,8-dioxatricyclo[5.1.0.02,4]octane ^a^	2.1	1656	1654	α-Cadinol	0.7
1287	1287	Safrole	0.7			Monoterpene hydrocarbons	40.0
1307	1314	Car-3-en-5-one	0.6			Oxygenated monoterpenoids	10.6
1314	1318	3-Hydroxycineole	2.3			Sesquiterpene hydrocarbons	27.9
1338	1338	δ-Elemene	2.0			Oxygenated sesquiterpenoids	8.8
1374	1376	α-Copaene	0.4			Others	1.4
						Total identified	88.6

RI_calc_: Retention index calculated with respect to a homologous series of *n*-alkanes on a ZB-5 column. RI_db_: Retention index from the databases. tr: trace (concentration < 0.05%). Major components (>5%) are highlighted in **bold**. ^a^ A reference RI was not available; the identification is tentative. ^b^ MS(EI): 220(12%), 202(9%), 107(10%), 199(50%), 159(44%), 149(26%), 135(32%), 133(23%), 123(82%), 121(30%), 119(50%), 117(20%), 109(36%), 107(49%), 105(41%), 94(40%), 93(75%), 91(89%), 81(64%), 80(100%), 79(85%), 77(48%), 69(41%), 67(27%), 55(32%), 43(79%), 41(45%).

**Table 2:** Reported studies on the chemical composition of essential oil from leaves of *Schinus terebinthifolia* (organized alphabetically by country).

Region	Number of Compounds	Main Compounds ^a^	Reference
Brazil (Bahia)	20	α-Pinene (22.3%), β-pinene (9.6%), limonene (16.2%), and α-copaene (6.3%)	[19]
Brazil (Caxias do Sul)	32	α-Pinene (38.7%), α-phellandrene (16.4%), β-pinene (12.3%), and limonene (8.5%)	[20]
Brazil (Espírito Santo)	21	Limonene (17.0%), β-pinene (14.6%), α-pinene (9.8%), α-copaene (7.9%), and germacrene D (8.8%)	[19]
Brazil (Mato Groso do Sul)	20	α-Pinene (14.9%), limonene (13.0%), α-copaene (10.2%), and β-pinene (6.6%)	[19]
Brazil (Minas Gerais)	47	Germacrene D (33.8%), (*E*)-β-caryophyllene (12.3%), and β-pinene (5.2%)	[22]
Brazil (North-East)	33	*p*-Cymen-7-ol (22.5%), 9-*epi*-(*E*)-caryophyllene (10.1%), carvone (7.5%), and verbenone (7.4%)	[23]
Brazil (Pará State)	52	Limonene (43.6%), δ-3-carene (27.6%), and (*E*)-β-caryophyllene (11.1%)	[15]
Brazil (Paraná)	21	Limonene (14.0%), α-pinene (11.4%), α-copaene (9.0%), β-pinene (8.6%), and germacrene D (6.9%)	[19]
Brazil (Pernambuco)	40	(*E*)-β-Caryophyllene (17.2%), aromadendrene (15.5%), bicyclogermacrene (8.6%), and *iso*-sylvestrene (7.4%)	[24]
Brazil (Porto Velho)	37	Germacrene D (25.0%), (E)-β-caryophyllene (17.5%), β-pinene (5.4%), and trans-β-elemene (5.3%)	[25]
Brazil (Rio Grande do Sul)	20	Limonene (19.5%), α-pinene (13.4%), α-copaene (9.1%), and β-pinene (8.5%)	[19]
Brazil (Rio Grando do Sul)	27	Limonene (14.2%), germacrene D (11.5%), α-copaene (8.0%), and α-pinene (7.9%),	[26]
Brazil (Rio Grando do Sul)	19	α-Pinene (21.6%), α-copaene (15.2%), and (*E*)-β-caryophyllene (13.8%)	[27]
Brazil (Sao Paulo)	16	α-Phellandrene (20.1%), limonene (11.7%), α-pinene (8.5%), and (E)-β-caryophyllene (6.8%)	[19]
Brazil (São Paulo)	49	Germacrene D (23.8%), biclyclogermacrene (15.0%), β-pinene (9.1%), β-longipinene (8.1%), and α-pinene (5.7%)	[14]
Brazil (Umuarama)	22	γ-Gurjunene (16.9%) and (*E*)-β-caryophyllene (16.0%), germacrene D (12.0%), α-pinene (11.6%), β-pinene (5.7%), and α-copaene (5.3%)	[13]
Brazil (Vitória)	32	δ-3-Carene (68.8%), and (*E*)-β-caryophyllene (8.2%)	[28]
Egypt (Kalubia Delta)	49	*cis*-β-Terpineol (17.9%), (*E*)-β-caryophyllene (17.6%), β-cedrene (9.8%), and citronellal (7.0%)	[12]
Pakistan	48	α-Pinene (15.5%), limonene (14.0%), α-phellandrene (12.4%), *p*-cymene (11.5%), and spathulenol (8.4%)	[11]
Tunisia (Al Ghazala)	88	α-Phellandrene (33.1%), α-pinene (14.2%), and limonene (6.6%), bicyclogermacrene (5.7%), and germacrene D (5.3%)	[29]
Tunisia (Medenine)	ND ^b^	Germacrene D (18.7%), α-phellandrene (16.9%), limonene (11.2%), and α-pinene (8.7%)	[21]

^a^ Components > 5%. ^b^ Only major components were reported.

**Table 3:** Antileishmanial activity against intracellular amastigote (IC_50_) and cytotoxicity effects on peritoneal macrophages from BALB/c mice (CC_50_) of the essential oil from leaves of *Schinus terebinthifolia* and pentamidine.

Product	IC_50_ ± SD (µg/mL)	CC_50_ ± SD (µg/mL)	Selectivity Index
EO-St	15.0 ± 1.6 *	53.4 ± 4.4 *	4
Pentamidine^®^	1.3 ± 0.1	11.7 ± 1.7	9

IC_50_: Median inhibitory concentration. CC_50_: Median cytotoxic concentration. SD: Standard deviation. Selectivity Index: CC_50_/IC_50_. * Statistical differences between EO-St and pentamidine (*p* < 0.05).

## Data Availability

All data are available in the article.

## Abbreviations

The following abbreviations are used in this manuscript:

CC_50_	Median Cytotoxic Concentration
CL	Cutaneous leishmaniasis
DMSO	Dimethylsulfoxide
DNP	2,4-Dinitrophenol
EOs	Essential oils
EO-St	Essential oil from *Schinus terebinthifolia*
GC-MS	Gas chromatography with mass spectrometry
HFBS	Heat-inactivated fetal bovine serum
IC_50_	Median Inhibitory Concentration
MDA	Malondialdehyde groups
MMP	Mitochondrial membrane potential
MPM	Murine peritoneal macrophage
MTT	3-[4,5-Dimethylthiazol-2-yl]-2,5-diphenyltetrazolium bromide
NBG	National Botany Garden
NTDs	Neglected Tropical Diseases
PP	Potential of peroxidation
RI	Retention index
ROS	Reactive oxygen species
SH	Sulfhydryl groups
tr	Trace
VL	Visceral leishmaniasis
